# Metastatic polyp of the gallbladder from renal cell carcinoma

**DOI:** 10.1186/s12885-017-3243-3

**Published:** 2017-04-04

**Authors:** Bor-Uei Shyr, Shih-Chin Chen, Yi-Ming Shyr, Rheun-Chuan Lee, Shin-E Wang

**Affiliations:** 1 Division of General Surgery, Department of Surgery, Taipei Veterans General Hospital and National Yang Ming University, 10 F 201 Section 2 Shipai Road, Taipei, 112 Taiwan; 2 Departments of Radiology, Taipei Veterans General Hospital, National Yang Ming University, Taipei, Taiwan

**Keywords:** Gallbladder, Metastasis, Renal cell carcinoma

## Abstract

**Background:**

Gallbladder metastasis from renal cell carcinoma (RCC) is extremely rare. The purpose of this study is to clarify the characteristics of metastatic RCC to gallbladder.

**Methods:**

The pooled data for analysis were collected from the case of metastatic RCC to gallbladder encountered by our institution along with sporadic cases reported in literature from 1991 to 2015.

**Results:**

A total of 50 cases of metastatic RCC to gallbladder were recruited for study. Fifty-seven percentage of the primary RCC was from the right kidney and 43% from the left. The median interval between diagnoses of primary and metastatic RCC to gallbladder was 36 months, with the longest duration up to 324 months. Most (70%) were asymptomatic. The size of metastatic RCC to gallbladder ranged from 0.8 cm to 9 cm, with median of 2.6 cm. Majority (91%) of the metastatic RCCs presented as a polypoid mass with narrow stalk, and 82% were hypervascular lesion. The overall 1 year, 3 year and 5 year survival rate was 91.5%, 76.2% and 59.3% respectively, with a median of 26.5 months. Number of the metastatic site, timing of gallbladder metastasis, symptom, tumor size and operation type of cholecystectomy seemed to have no impact on survival.

**Conclusions:**

Metastatic RCC to the gallbladder should be taken into account for a gallbladder polypoid mass with narrow hypervascular stalk during the diagnosis and/or follow-up of primary RCC. Gallbladder metastasis from RCC is not necessarily to be an advanced stage with poor outcome, and cholecystectomy is recommended whenever possible.

## Background

Metastasis to the gallbladder has been considered rare, and reported from a variety of primary sites, it usually manifests at a late and advanced stage of malignancy [1–3]. It is estimated that 30% - 40% of patients with RCC have already had synchronous metastasis at time of presentation and another 20% - 50% will develop metachronous metastasis after nephrectomy for the primary RCC [2, 4–6]. The common sites of RCC metastasis are lung, bone, liver, brain, adrenal and contralateral kidney. Gallbladder metastasis from RCC has been reported sporadic, and considered extremely rare [7–10]. it is usually detected only at autopsy with a rate of less than 0.6% [10]. Clinical diagnosis of gallbladder metastasis is even rarer [3, 11, 12, 5, 7, 8]. Patients with distant metastasis from RCC usually present a poor prognosis, with a 5-year survival rate of <10%. Nevertheless, it is well-known that complete resection of metastasis could have long-term survival in selected patients with pancreas metastasis of RCC [13]. This favorable prognosis might also be the case for those with gallbladder metastasis [11].

The purposes of this article are to present our clinical experience with metastatic polyp of the gallbladder from RCC and to analyze an expanded sample size by adding cases reported in the literature to our pool of the study cases. Thus, a statistic attempt is made to clarify the demographics, clinical presentations, managements and survival outcomes of this rare tumor.

## Methods

Brief description was made for the case of metastatic polyp of the gallbladder from RCC encountered at our institution. The study has been approved by the Institutional Review Board of Taipei Veterans General Hospital (IRB-TPEVGH No.: 2017-03-006BAC). Appropriate written informed consent to participate was obtained from the patients. Data and materials described in the manuscript, including all relevant raw data, will be freely available to any scientist wishing to use them for non-commercial purposes, without breaching participant confidentiality. To clarify the characteristics of metastatic RCC to gallbladder, individualized data of these cases described in the English literature were extracted and added to our database to expand the study sample size for a more complete analysis. Two methods were utilized to search for relevant cases in the literature. First, to identify the relevant articles dealing with metastatic RCC to gallbladder in the English literature, a computerized search was performed on the PubMed electronic database, covering data from 1991 to 2015. The following keywords were used for the PubMed search: metastatic renal cell carcinoma and gallbladder, gallbladder metastasis and renal cell carcinoma, renal cell carcinoma metastasis and gallbladder. Second, the reference lists of PubMed-selected metastatic RCC to gallbladder articles were screened systematically for additional studies of interest. A total of 35 related articles were selected for study [1–5, 7–9, 11, 12, 14–38].

Cases without individualized data and duplicate cases reported in literature were excluded from the analysis. The data pool from the related literature cases and our patient were analyzed to determine the characteristics of metastatic RCC to gallbladder including demographics, primary renal cell carcinoma, clinical presentations of RCC metastasis to gallbladder, timing of metastasis, concomitant metastasis to other site, and survival outcomes whenever possible.

Statistical analyses were performed using Statistical Product and Service Solutions (SPSS) version 21.0 software (SPSS Inc., IBM, Armonk, NY, USA). All continuous data were presented as median (range) and mean ± standard deviation (SD), and case number (%) were presented when appropriate to the type of data. Actuarial survival was estimated via the Kaplan–Meier method, and a log rank test was used to determine differences in the subgroups. For all analyses, a *P* value less than 0.050 was considered statistically significant.

## Results

**Fig. 1:**
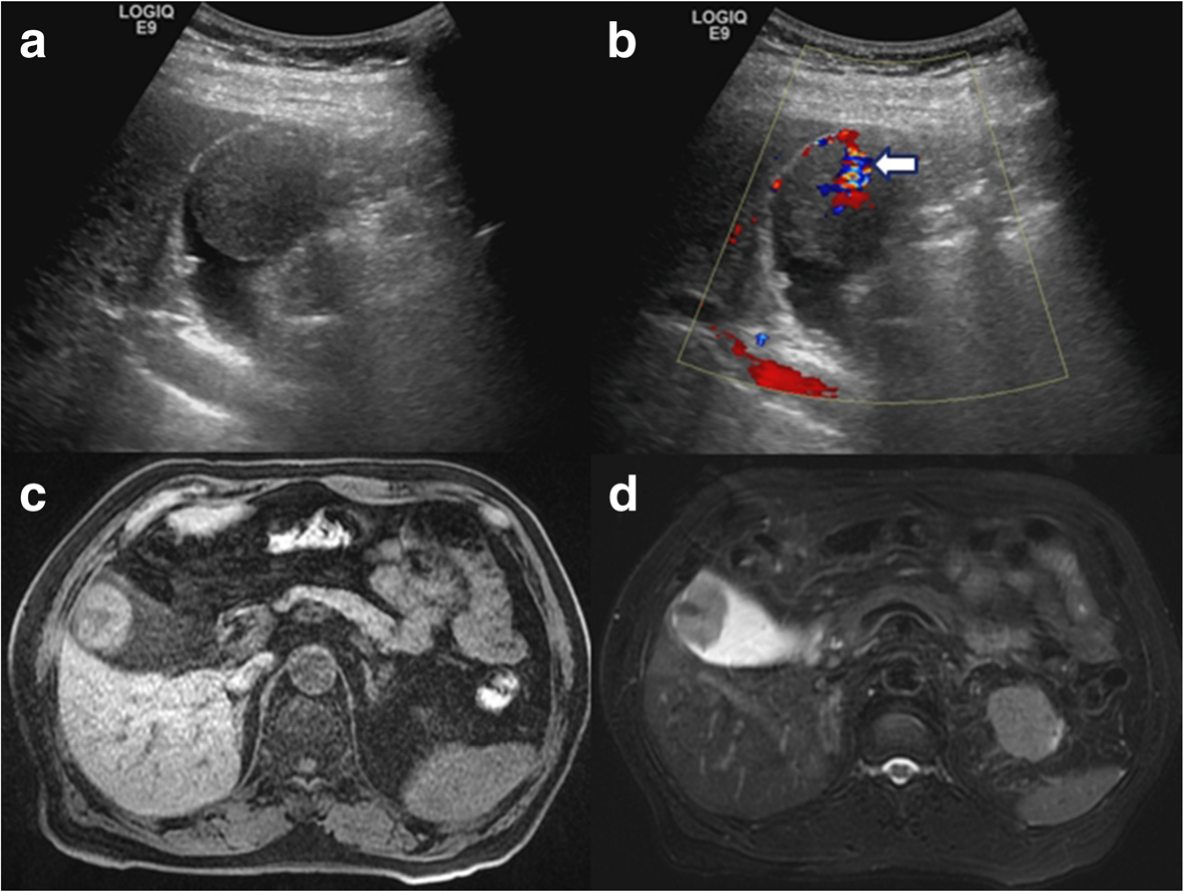
The *gray-scale* (**a**) and color Doppler sonography (**b**) show arterial flow (*white arrow*) within the polypoid mass from the cystic artery. On the T1-weighted fat-saturated spoiled gradient recalled echo (**c**) and fat-saturated T2 fast spin-echo MRI images (**d**), the polypoid gallbladder tumor demonstrates intermediate signal intensity on T1-weighted image and slightly high on T2-weighted image to that of liver parenchyma

**Fig. 2:**
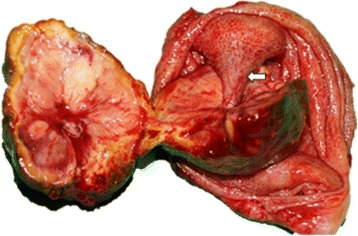
The metastatic renal cell carcinoma presents a well-circumscribed polypoid mass with a narrow stalk (*white arrow*) to the fundus of gallbladder

A total of 50 cases of metastatic RCC to the gallbladder were collected for study, including 49 cases from the literature and 1 from our institution. Our case, an 80-year-old man, was diagnosed with a gallbladder tumor during a postoperative surveillance follow-up by sonography which showed a big hypoechoic 3.6 × 3.7 cm mass (Fig. 1a) with a hypervascular stalk (Fig. 1b) to gallbladder fundus in November 2011. The patient had a history of radical nephrectomy for right RCC with stage of pT1aN0M0 in November 1997. Magnetic resonance imaging (MRI) revealed a 4.2 × 3.4 cm pedunculated polypoid lesion arising from gallbladder fundus. The polypoid gallbladder tumor demonstrated intermediate signal intensity on T1-weighted image (Fig. 1c), slightly high on T2-weighted image (Fig. 1d), and high intensity on diffusion-weighted image. Serum tumor markers including alpha-fetoprotein (AFP), carbohydrate antigen 19-9 (CA 19-9) and carcinoembryonic antigen (CEA) were all within normal limit. The patient underwent an open cholecystectomy under the impression of gallbladder polyp with malignant change in March 2012. The resected specimen showed a big well-circumscribed polypoid mass with a narrow stalk (Fig. 2) attached to the gallbladder fundus, and the turned out to be a metastatic RCC by pathologic examination. The patient recovered uneventfully and remained disease-free without further adjuvant therapy for 3.5 years.

Analysis of the pooled data (Table 1) indicated that 63% patients were male and 37% were female. The metastatic RCC to the gallbladder occurred at a median age of 62 years, ranging from 39 to 80 years. Fifty-seven percentage of the primary RCC was from the right kidney and 43% from the left. Size of primary RCC ranged from 0.4 cm to 12.2 cm with a median of 7.8 cm. Majority (75%) of RCC metastasis to the gallbladder were metachronous. The median interval between diagnoses of primary and metastatic RCC to gallbladder for the metachronous metastasis was 60 months, with the longest duration up to 324 months. Most (70%) were asymptomatic, while epigastric pain was the most common presentation 23% in symptomatic patients. The polypoid mass in gallbladder was found by sonography in 93%, by computed tomography (CT) in 80%, and by magnetic resonance imaging (MRI) in 21%.

**Table 1:** Demographics and clinical presentations of gallbladder metastasis from RCC

Sex, *n* = 40	
Male	25 (63%)
Female	15 (37%)
Age, y/o, *n* = 39	
Median (range)	62 (39 – 80)
Mean’s	61 ± 12
Laterality of primary RCC, *n* = 42	
Right	24 (57%)
Left	18 (43%)
Size of primary RCC, cm, *n* = 20	
Median (range)	7.8 (0.4 – 12.2)
Mean ± SD	7.2 ± 3.3
Timing of gallbladder metastasis, *n* = 48	
Synchromous	12 (25%)
Metachronous	36 (75%)
Interval of metachronous metastasis, month, *n* = 33	
Median (range)	60 (1 – 324)
Mean ± SD	79.4 ± 58.2
Symptoms, *n* = 43	
No	30 (70%)
Epigastric pain	10 (23%)
Nausea or vomiting	4 (9%)
Epigastric fullness	3 (7%)
Body weight loss	2 (5%)
Jaundice	1 (2%)
Duration of symptom, month, *n* = 37	
Median (range)	0 (0 – 6)
Mean ± SD	0.3 ± 1.0
Diagnosis method	
Sonography, *n* = 42	39 (92.9%)
CT scan, *n* = 44	35 (79.5%)
MRI, *n* = 39	8 (20.5%)

*RCC* renal cell carcinoma, *SD* standard deviation, *MRI* magnetic resonance imaging, *CT* computed tomography

Table 2 described the diagnosis and characteristics of gallbladder metastasis from RCC. Preoperative diagnosis suspected metastatic RCC to gallbladder in 44%, gallbladder polyp in 27% and gallbladder cancer in 17%. The size of metastatic RCC to gallbladder ranged from 0.8 cm to 9 cm, with median of 2.6 cm. Majority (91%) of the metastatic RCCs presented as a polypoid mass with narrow stalk, and 82% were hypervascular lesion by image studies. The metastatic RCC was located on gallbladder fundus in 48%, body in 41% and neck in 12%. Most (72%) of the metastatic RCCs to the gallbladder were not associated with gallstone. Multiple metastasis occurred in 28% at the diagnosis of metastatic RCC to gallbladder, and the most common concomitant additional site of RCC metastasis was contralateral kidney and lung (12.8%), followed by bone (6.4%).

**Table 2:** Diagnosis and characteristics of gallbladder metastasis from RCC

Preoperative diagnosis, *n* = 41	
Gallbladder metastasis from RCC	18 (44%)
Gallbladder polyp	11 (27%)
Gallbladder cancer	7 (17%)
Acute cholecystitis	3 (7%)
Gallstone	2 (5%)
Size of metastatic RCC to gallbladder, cm, *n* = 40	
Median (range)	2.6 (0.8 – 9)
Mean ± SD	2.9 ± 1.8
Vascularity of metastatic RCC to gallbladder, *n* = 27	
Hypervascular	22 (82%)
Heterogenous	5 (18%)
Location of metastatic RCC in gallbladder, *n* = 42	
Fundus	20 (48%)
Body	17 (40%)
Neck	5 (12%)
Appearance of metastatic RCC in gallbladder, *n* = 42	
Polypoid mass with narrow stalk to gallbladder	38 (91%)
Polypoid mass with wide base to gallbladder	4 (9%)
Presence of gallbladder stone, *n* = 39	
No	28 (72%)
Yes	11 (28%)
Concomitant multiple metastasis, *n* = 47	13 (28%)
Contralateral kidney	6 (13%)
Lung	6 (13%)
Bone	3 (6%)
Adrenal	2 (4%)
Pancreas	1 (2%)

*RCC* renal cell carcinoma, *SD* standard deviation

Eighty-seven percentage of patients were treated with cholecystectomy by open laparotomy, 13% by laparoscopic approach, and 25% received additional adjuvant therapy. Two-thirds of patients had no recurrence. The overall 1 year, 3 year and 5 year survival was 91.5%, 76.2% and 59.3% respectively, with a median of 26.5 months (1–132 months) (Table 3). Number of the metastatic site, timing of metastasis gallstone, symptom, tumor size and operation type of cholecystectomy seemed to have no survival impact.

**Table 3:** Survival outcomes of gallbladder metastasis from RCC

	Median (range)	1 year survival	3 year survival	5 year survival	*P* value
	Mean ± SD, month				
Overall, *n* = 26	26.5 (1 – 132)	91.5%	76.2%	59.3%	
	35.3 ± 31.4				
Gallbladder metastasis					0.333
Solitary, *n* = 15	27 (6 – 96)	93.3%	80.0%	80.0%	
	40.6 ± 26.4				
Combined with other site metastasis, *n* = 11	12 (1 – 132)	87.5%	70.0%	23.3%	
	28.1 ± 37.4				
Gall stone					0.831
Without, *n* = 15	24 (1 – 96)	92.3%	76.9%	46.2%	
	30.4 ± 25.9				
With, *n* = 6	26 (6 – 47)	100%	66.7%	66.7%	
	25.5 ± 15.1				
Symptom					0.317
Without, *n* = 19	26 (1 – 132)	94.4%	84.0%	60.0%	
	36.9 ± 32.0				
With, *n* = 6	18.5 (3 – 96)	75.0%	50.0%	50.0%	
	31.5 ± 36.0				
Timing of metastasis					0.325
Synchronous, *n* = 8	25 (1 – 132)	85.7%	85.7%	85.7%	
	38 ± 42.1				
Metachronous, *n* = 17	27 (3 – 96)	93.3%	70.0%	46.7%	
	33.4 ± 27.4				
Tumor size					0.607
≤ 2 cm, *n* = 9	37 (6 – 132)	88.9%	74.1%	44.4%	
	42.4 ± 37.7				
> 2 cm, *n* = 16	24 (1 – 96)	92.3%	76.9%	76.9%	
	31.9 ± 29.0				
Cholecystectomy					0.398
Open, *n* = 23	27 (1 – 132)	90.5%	74.0%	55.5%	
	37.1 ± 32.4				
Laparoscopic, *n* = 3	12 (6 – 47)	100%	100%	100%	
	21.7 ± 22.1				

## Discussion

Metastatic gallbladder tumors are not common, about 4.8% of all gallbladder malignancies [39]. melanoma, stomach cancer, RCCs, hepatocellular carcinoma, colorectal cancer, breast cancer, and lung cancers have been reported to be the common cancers that could metastasize to the gallbladder [3, 4, 39]. RCC, accounting for 3% of all malignancies in adults and 85% of primary renal tumors, has a great propensity to develop synchronous or metachronous metastasis to various anatomic sites [1, 40]. Moreover, late and solitary metastasis have been a unique clinical presentation for RCC after nephrectromy [13].

This study found gallbladder metastasis from RCC occurred with male predominance (63%), and at a median age of 62 years. Although the gallbladder is in right abdomen, primary RCC could be from either the right (57%) or left kidney (43%). Most (75%) of gallbladder metastases from RCC were metachronous. The median time to gallbladder metastasis following nephrectomy in metachronous cohort was 5 years, with longest one up to 27 years. Majority (72%) were solitary gallbladder metastasis without other site metastasis at the time of diagnosis. The unique clinical entity of solitary and late metachronous metastasis seems to be observed not only in other organs such as pancreas [13] but also in gallbladder. Although the median tumor size was 2.6 cm, most (70%) of the patients remained asymptomatic at the time of diagnosis and/or follow-up of primary RCC. The low association (28%) of gallstone might explain the silent clinical presentation.

Although gallbladder polyp (27%), gallbladder cancer (17%) and acute cholecystitis (7%) were considered in some patients preoperatively, metastatic RCC to the gallbladder was nevertheless on the priority diagnosis in 44% cases with the history of primary RCC. In this study, we found most (91%) of the metastatic RCCs presented as a polypoid mass with narrow stalk, and 82% were hypervascular lesion by image studies. It has been observed that metastatic adenocarcinoma of the GB manifested as infiltrative and persistently enhancing wall thickenings, while the metastatic non-adenocarcinoma such as RCC usually manifested as a polypoid lesion in the gallbladder by computed tomography (CT) imaging study [3, 4, 14]. Some imaging studies with ultrasound, CT scan or MRI also showed a hypervascular polypoid mass like that of our case [4, 14]. The metastatic polypoid mass showed high intensity on diffusion-weighted images, and the apparent diffusion coefficient was relatively low on MRI. These imaging findings are considered characteristic and may be helpful for preoperative diagnosis [4, 14]. Therefore, metastatic RCC should be taken into account for a gallbladder polyp with narrow hypervascular stalk, especially for those with the history of RCC.

Gallbladder metastasis from RCC is not necessarily to be an advanced stage with poor outcome, especially for those with late and solitary metastasis [4, 7, 12, 13]. Based on the experience in the more typical metastatic sites such as pancreas, lung bone, resectability, long time to recurrence, good performance status and oligometastatic disease have better benefit of metastasectomy [10, 13, 41]. Cholecystectomy should be considered because only such management could provide an opportunity for longer survival [16, 25–27, 34]. This study revealed the median survival time was 26.5 months, with 91.5% 1-year and 59.3% 5-year survival. A multivariate analysis showed that favorable predictors of survival for metastatic RCC included single site of first recurrence, curative resection of first metastasis, long disease-free interval, solitary site of first metastasis, and metachronous metastasis [42]. It is also shown that the longer interval between diagnosis of RCC and gallbladder metastasis the better the survival [15]. In this study, number of the metastatic site, timing of metastasis gallstone, symptom, tumor size and operation type of cholecystectomy seemed to have no survival impact. However, it is hard to draw a solid conclusion from this retrospective study with a small sample size.

## Conclusions

Gallbladder metastasis from RCC is a rare but unique clinical entity. Metastatic RCC should be taken into account for a gallbladder polypoid mass with narrow hypervascular stalk at the diagnosis and/or follow-up of primary RCC. Gallbladder metastasis from RCC is not necessarily to be an advanced stage with poor outcome, especially for those with late and solitary metastasis. Cholecystectomy should be considered to determine the definitive diagnosis and provide better survival outcome whenever possible.

## Abbreviations

AFP: Alpha-fetoprotein
CA 19-9: Carbohydrate antigen 19-9
CEA: Carcinoembryonic antigen
CT: Computed tomography
MRI: Magnetic resonance imaging
RCC: Renal cell carcinoma
